# Changes in Serum Bone Metabolism Markers after Living Donor Liver Transplantation (LDLT) and Their Association with Fracture Occurrences

**DOI:** 10.3390/life13071438

**Published:** 2023-06-25

**Authors:** Shu-Jui Kuo, Chao-Long Chen, Sung-Hsiung Chen, Jih-Yang Ko

**Affiliations:** 1 School of Medicine, China Medical University, Taichung 404328, Taiwan; b90401073@gmail.com; 2 Department of Orthopedic Surgery, China Medical University Hospital, Taichung 404327, Taiwan; 3 Department of Surgery, College of Medicine, Chang Gung University, Kaohsiung Chang Gung Memorial Hospital, Kaohsiung 833401, Taiwan; clchen@cgmh.org.tw; 4 Department of Orthopedic Surgery, College of Medicine, Chang Gung University, Kaohsiung Chang Gung Memorial Hospital, Kaohsiung 833401, Taiwan; chensh@cgmh.org.tw; 5 Center for Shockwave Medicine and Tissue Engineering, College of Medicine, Chang Gung University, Kaohsiung Chang Gung Memorial Hospital, Kaohsiung 833401, Taiwan

**Keywords:** living donor liver transplantation (LDLT), Dickkopf-related protein 1 (DKK-1), osteoprotegerin (OPG), nuclear factor kappa B ligand (RANKL)

## Abstract

Living donor liver transplantation (LDLT) is lifesaving, but can lead to osteoporosis and fractures. In our 3-year study of 25 LDLT recipients, we observed significant reductions in lumbar spine and femoral neck T scores, along with bone resorption marker reductions and liver regeneration marker increases. Serum calcium levels increased, while osteoprotegerin (OPG) decreased and Dickkopf-related protein 1 (DKK-1) increased. Patients who suffered fractures within 3 years of LDLT had higher serum OPG, lower serum nuclear factor kappa B ligand (RANKL), a higher OPG/RANKL ratio and higher serum DKK-1 levels. OPG, RANKL, OPG/RANKL ratio and DKK-1 levels before LDLT predicted hip or spine fractures within three years after LDLT. Further research is necessary to determine the optimal level of osteoclastic activity for preventing fracture onset.

## 1. Introduction

Living donor liver transplantation (LDLT) is a life-saving operation with a good long-term survival rate, a biliary complications rate < 7% and a reoperation rate < 3% [1,2,3,4,5]. Due to the merits mentioned above, LDLT is the most common form of liver transplantation in Asia at present [6]. However, osteoporosis and subsequent fracture remain the major complications threatening the life quality and long-term survival of the recipients [7]. The risk factors of post-transplant bone loss include the exposure to steroid and other immunosuppressive agents, secondary hyperparathyroidism, hypogonadism, vitamin D deficiency and pre-existing bone disease [7]. However, the underlying mechanisms have remained obscure until now and the treatments are still empiric and unsatisfactory. There is currently no indicator to predict the occurrence of fracture after LDLT.

In this study, we aimed to investigate bone metabolism after LDLT in view of the interplay of multiple bone turnover markers, thus unraveling the multi-faceted mechanisms leading to postoperative bone loss. We also tried to determine the pre-LDLT parameters that could predict the occurrence of fracture after LDLT.

## 2. Materials and Methods

Twenty-five patients (twenty male and five female) with end-stage liver disease undergoing LDLT at Kaohsiung Chang Gung Memorial Hospital between January 2009 and December 2009 were enrolled. This study was approved by the Institutional Review Board of Kaohsiung Chang Gung Memorial Hospital (IRB 97-1233B). All participants signed a valid written informed consent form before enrollment. Exclusion criteria included renal dysfunction (glomerular filtration rate < 60 mL per hour), use of anti-osteoporotic agents and history of hip or spine fracture before the surgery. The donors were all adult relatives of the patients. All donors who participated in this study were not from any vulnerable population, and each of them voluntarily provided written informed consent.

As for immunosuppressive agents, prednisolone 5–20 mg per day was prescribed initially, and then tapered off within 6 months. Mycophenolate mofetil 250 mg twice per day and tacrolimus 1 mg twice per day were given right after LDLT, then tapered down gradually to the lowest dosage possible to maintain long-term immunosuppression.

All patients received daily calcium and vitamin D supplementation in accordance with the recommendation of the National Institutes of Health (NIH) (http://ods.od.nih.gov/) (accessed on 1 January 2009), and they were strongly encouraged to adhere to the prescribed regimen. This supplementation regimen was initiated prior to liver transplantation and continued every three months for a duration of one year at the clinic of the corresponding author. None of the patients were given anti-resorptive or bone formation-stimulating agents during participation in the study.

Bone mineral density (BMD) and T score values in the lumbar spine and hip were detected using dual-energy X-ray absorptiometry (DEXA) (Hologic ODR 4500A, Waltham, MA, USA) before (0M) and at three (3M), six (6M) and twelve (12M) months after LDLT.

All the patients were followed for the occurrence of hip or spine fractures for at least three years after LDLT.

Medical and rejection-related conditions were closely monitored throughout the study, and liver biopsy was arranged if clinical suspicions arose regarding their deterioration. Five milliliters of peripheral vein blood were drawn from each patient with fasting for more than eight hours before and at 3, 6 and 12 months after LDLT. The samples were processed to collect serum and then stored at −80 °C until analysis. The bone formation markers analyzed included bone alkaline phosphatase (BAP) and osteocalcin (OCN) (Invitrogen, Waltham, MA, USA.) The bone resorption markers measured include tartrate-resistant acid phosphatase (TRAP) (ASIA bioscience Co. Taipei city, Taiwan) and carboxyterminal telopeptide of type I procollagen (CTX). Liver regeneration markers included insulin-like growth factor-I (IGF-I) and 25 hydroxyvitamin D (25-OH-D). The intact parathyroid hormone (iPTH), calcium and phosphorus levels were measured to assess the mineral metabolism. Other markers analyzed include osteoprotegerin (OPG), receptor activator of nuclear factor kappa B ligand (RANKL) and Dickkopf-related protein 1 (DKK1). The markers mentioned above were detected using ELISA kits following the respective manufacturers’ instructions or detected by sending them to the department of laboratory medicine of Kaohsiung Chang Gung Memorial Hospital if the manufacturer was not mentioned.

The Friedman test was utilized for the assessment of repeated within-group measurements for continuous variables, and the Wilcoxon signed-rank test was applied for post hoc analysis [8]. We used Spearman correlation coefficient to measure the strength of correlation. All statistics were carried out using IBM SPSS software version 21.0 (IBM Corp. Armonk, NY, USA), and a *p* value of <0.05 was defined as statistically significant.

## 3. Results

The baseline demographic data are summarized in Table 1. None of the patients suffered from hip or spine fracture before LDLT.

**Figure 1:**
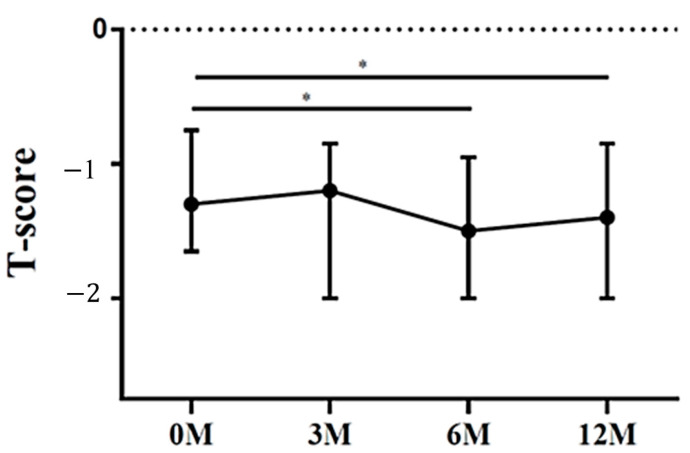
The changes in lumbar spine T score before and after LDLT. Friedman test *p* value: 0.024. Post hoc *p* values: 0M/6M 0.012, 0M/12M 0.045. * *p* < 0.05.

**Figure 2:**
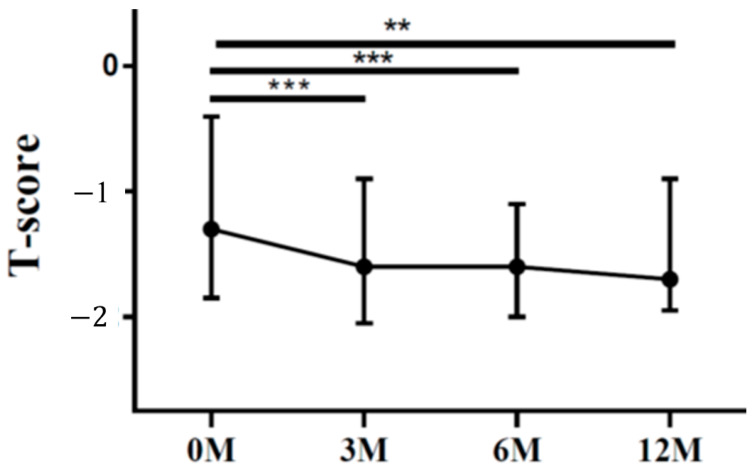
The changes in femoral neck T score before and after LDLT. Friedman test *p* value: <0.001. Post hoc *p* values: 0M/3M < 0.001, 0M/6M < 0.001, 0M/12M 0.001. ** *p* < 0.01, *** *p* < 0.001.

The lumbar spine T scores 6 months (*p* = 0.012) and 12 months (*p* = 0.045) after LDLT were all significantly lower than the preoperative baseline (Figure 1). The femoral neck T scores 3 months (*p* < 0.001), 6 months (*p* < 0.001) and 12 months (*p* = 0.001) after LDLT were all significantly lower than the preoperative baseline (Figure 2).

**Figure 3:**
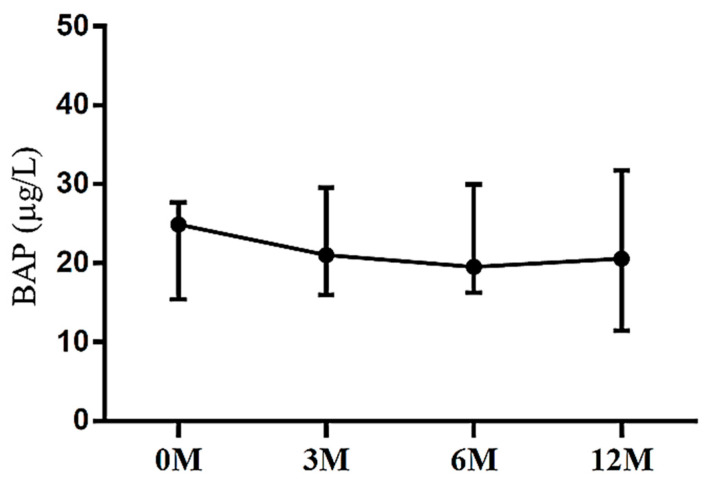
The changes in serum bone alkaline phosphatase (BAP) before and after LDLT. Friedman test *p* value: 0.577.

**Figure 4:**
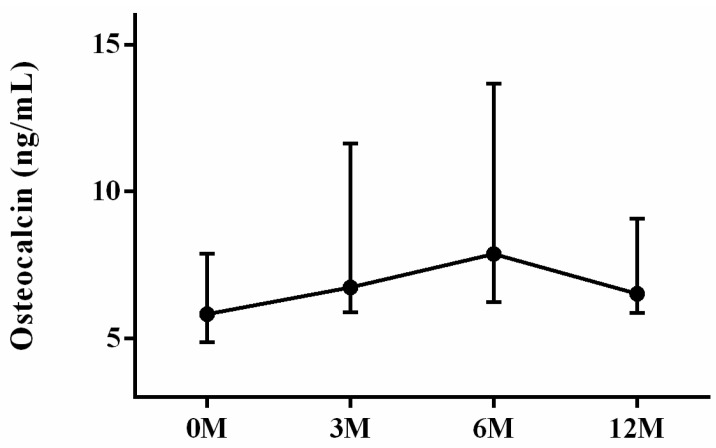
The changes in serum osteocalcin (OCN) before and after LDLT. Friedman test *p* value: 0.082.

Regarding bone formation markers, we assessed the levels of BAP and OCN. BAP is predominantly synthesized by osteoblasts and plays a crucial role in bone matrix mineralization. Elevated serum BAP levels are indicative of heightened systemic osteoblastic activity [9]. OCN is a prevalent non-collagenous protein found in bone tissue. It is synthesized by osteoblasts during the formation of osteoids and is subsequently released into the bloodstream to facilitate bone growth [9]. We showed that no apparent intra-group differences in BAP (Figure 3) and OCN (Figure 4) were noted (*p* = 0.577 and *p* = 0.082, respectively).

**Figure 5:**
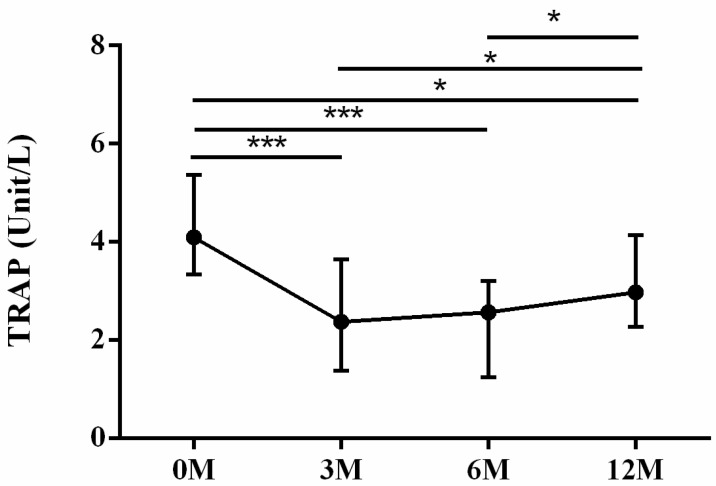
The changes in serum tartrate-resistant acid phosphatase (TRAP) before and after LDLT. Friedman test *p* value: <0.001. Post hoc *p* values: 0M/3M < 0.001, 0M/6M < 0.001, 0M/12M 0.046, 3M/12M 0.036, 6M/12M 0.012. * *p* < 0.05, *** *p* < 0.001.

**Figure 6:**
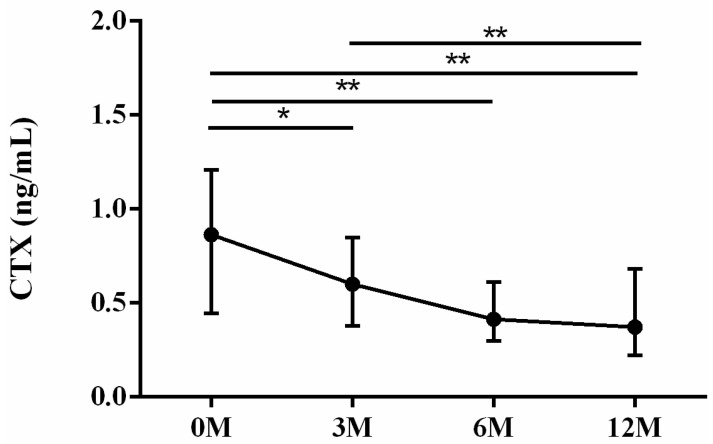
The changes in serum carboxyterminal telopeptide of type I procollagen (CTX) before and after LDLT. Friedman test *p* value: 0.001. Post hoc *p* values: 0M/3M 0.025, 0M/6M 0.001, 0M/12M 0.001, 3M/12M 0.019. * *p* < 0.05, ** *p* < 0.01.

As for bone resorption markers, tartrate-resistant acid phosphatase (TRAP) (Figure 5) and carboxyterminal telopeptide of type I procollagen (CTX) (Figure 6) were examined. TRAP is a protein majorly secreted by osteoclasts in bone resorption and excreted by urine. It could serve as an indicator of osteoclast activity and quantity [10]. CTX is derived from collagen degradation mediated by osteoclasts. CTX is frequently utilized to evaluate bone resorption activity and to monitor the effectiveness of drug therapy for osteoporosis [11]. The serum TRAP levels 3 months (*p* < 0.001), 6 months (*p* < 0.001) and 12 months (*p* = 0.046) after LDLT were all significantly lower than the preoperative baseline. The serum TRAP levels 3 months (*p* = 0.036) and 6 months (*p* = 0.012) after LDLT were all lower than the TRAP levels 12 months after LDLT (Figure 5). The serum CTX levels 3 months (*p* = 0.025), 6 months (*p* = 0.001) and 12 months (*p* = 0.001) after LDLT were all significantly lower than the preoperative baseline. The serum CTX levels 3 months (*p* = 0.019) after LDLT were higher than the CTX levels 12 months after LDLT (Figure 6).

**Figure 7:**
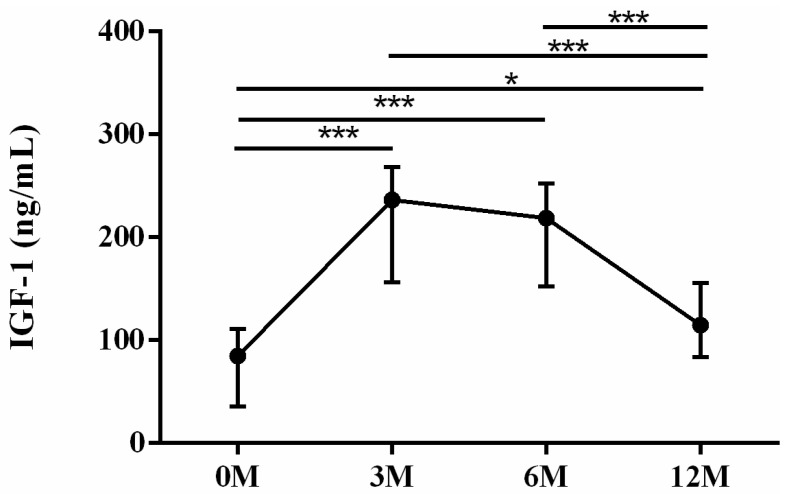
The changes in serum insulin-like growth factor-1 (IGF-1) levels before and after LDLT. Friedman test *p* value: <0.001. Post hoc *p* values: 0M/3M < 0.001, 0M/6M < 0.001, 0M/12M 0.011, 3M/12M < 0.001, 6M/12M < 0.001. * *p* < 0.05, *** *p* < 0.001.

**Figure 8:**
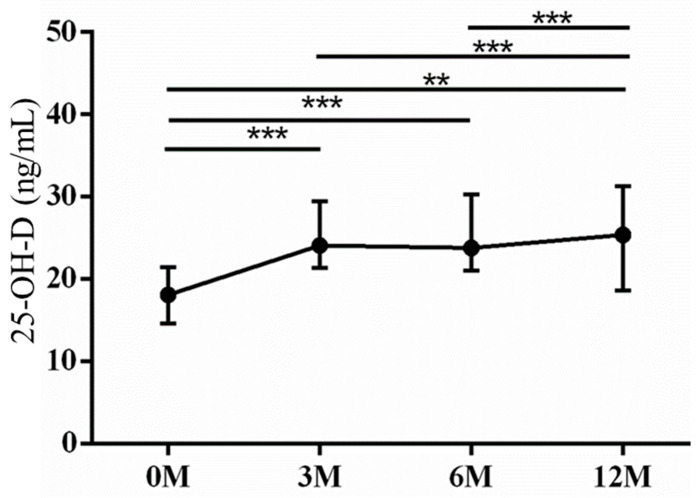
The changes in serum 25-OH-D levels before and after LDLT. Friedman test *p* value: 0.001. Post hoc *p* values: 0M/3M < 0.001, 0M/6M < 0.001, 0M/12M 0.001, 3M/12M < 0.001, 6M/12M < 0.001. ** *p* < 0.01 and *** *p* < 0.001.

As for the liver regeneration markers, insulin-like growth factor-1 (IGF-1) (Figure 7) and 25-OH-D (Figure 8) levels were assessed. Insulin-like growth factor 1 (IGF-1) is primarily synthesized in hepatocytes and exerts regulatory effects on bone metabolism, playing a significant role in bone growth and maintenance [12]. Patients with chronic liver disease complicated by osteoporosis exhibited reduced serum levels of IGF-1 compared with those without osteoporosis. Lower serum levels of IGF-1 were found to be associated with an increased risk of hip and vertebral fractures in older men [12]. Deficiency of 25-OH-D has been demonstrated to be associated with a higher incidence of bone fatigue and stress fractures [13,14]. The 3 month (*p* < 0.001), 6 month (*p* < 0.001) and 12 month (*p* = 0.011) serum IGF-1 levels were all higher than preoperative baseline. The 3 month (*p* < 0.001) and 6 month (*p* < 0.001) serum IGF-1 levels were all higher than the 12 month levels (Figure 7). The 3 month (*p* < 0.001), 6 month (*p* < 0.001) and 12 month (*p* = 0.001) serum 25-OH-D levels were all higher than preoperative baseline. The 3 month (*p* < 0.001) and 6 month (*p* < 0.001) serum 25-OD-D levels were all lower than the 12 month levels (Figure 8).

**Figure 9:**
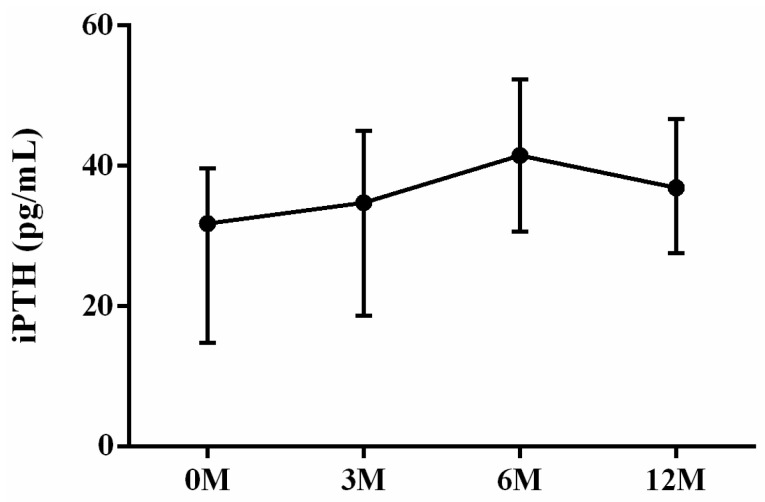
The changes in serum iPTH levels before and after LDLT. Friedman test *p* value: 0.054.

**Figure 10:**
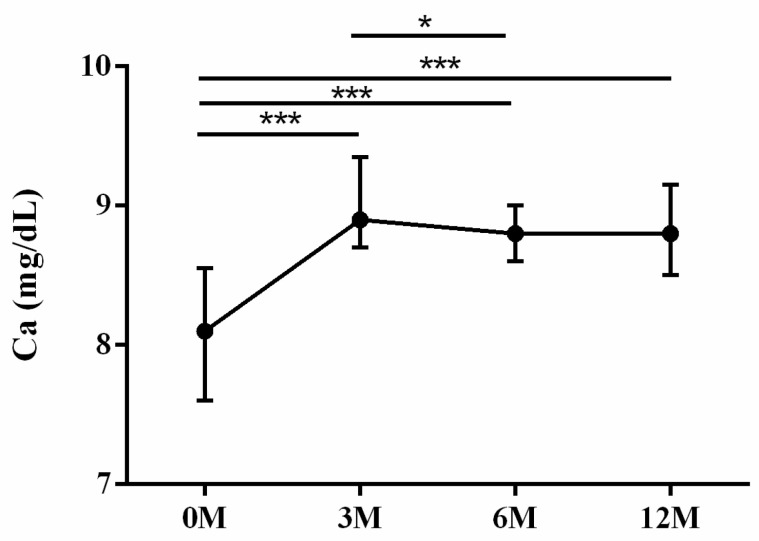
The changes in serum Calcium (Ca) levels before and after LDLT. Friedman test *p* value: <0.001. Post hoc *p* value: 0M/3M < 0.001, 0M/6M < 0.001, 0M/12M < 0.001, 3M/6M 0.038. * *p* < 0.05, and *** *p* < 0.001.

**Figure 11:**
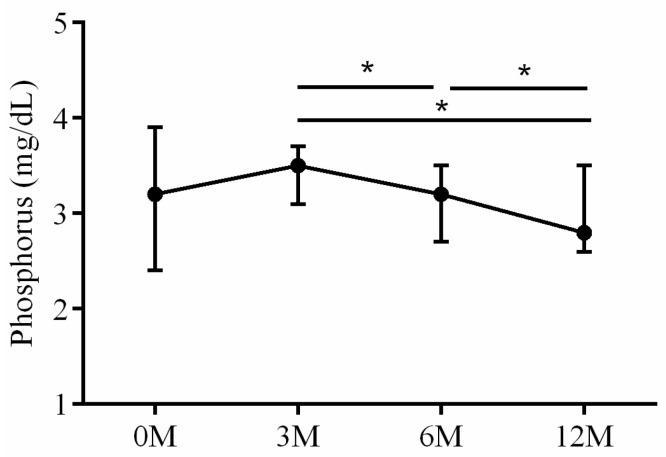
The changes in serum Phosphorus (P) levels before and after LDLT. Friedman test *p* value: 0.045. Post hoc *p* value: 3M/6M 0.023, 3M/12M 0.003, 6M/12M 0.039. * *p* < 0.05.

Calcium phosphate homeostasis is mainly regulated by vitamin D, iPTH and fibroblast growth factor 23 [15]. As for the mineral metabolism, intact parathyroid hormone (iPTH) (Figure 9), calcium (Figure 10) and phosphorus (Figure 11) were assessed. Serum iPTH levels did not demonstrate apparent changes before and after LDLT (*p* = 0.054). Serum calcium levels 3 months (*p* < 0.001), 6 months (*p* < 0.001) and 12 months (*p* < 0.001) after LDLT were all higher than preoperative baseline. The 3 month (*p* = 0.038) serum calcium levels were higher than the 6 month levels (Figure 10). The 6 month (*p* = 0.023) and 12 month (*p* = 0.003) serum phosphorus levels were all lower than the 3 month values. The 6 month (*p* = 0.039) serum phosphorus levels were higher than the 12 month values (Figure 11).

**Figure 12:**
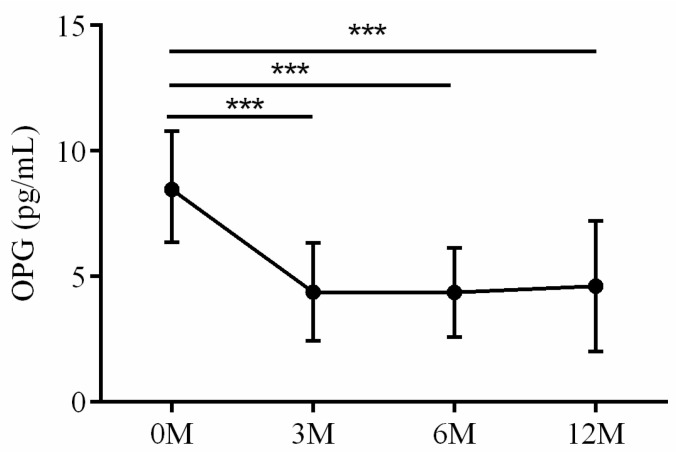
The changes in serum OPG before and after LDLT. Friedman test *p* value: 0.002. Post hoc *p* value: 0M/3M < 0.001, 0M/6M < 0.001 and 0M/12M < 0.001. *** *p* < 0.001.

**Figure 13:**
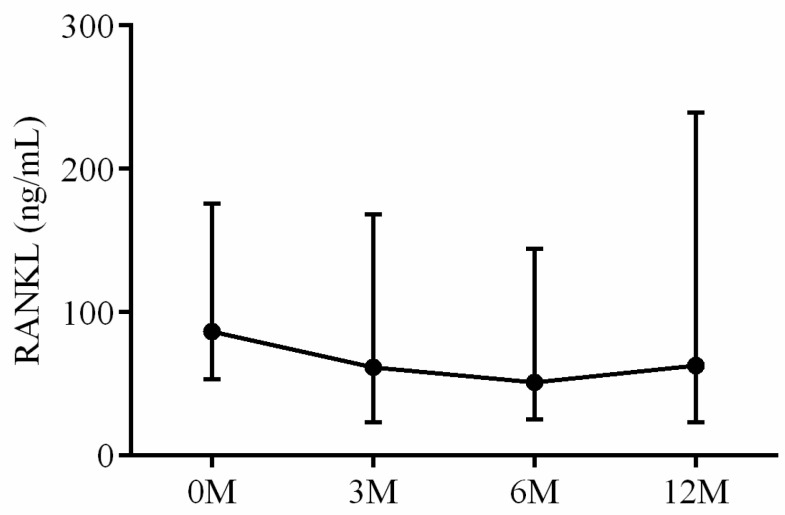
The changes in serum RANKL before and after LDLT. Friedman test *p* value: 0.744.

**Figure 14:**
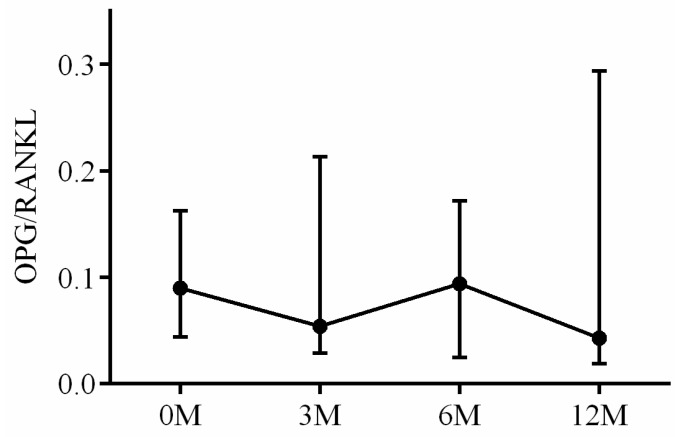
The changes in serum OPG/RANKL ratio before and after LDLT. Friedman test *p* value: 0.491.

The serum OPG, RANKL and OPG/RANKL ratio were also assayed. The interaction between RANKL and the receptor activator of nuclear factor kappa B (RANK) regulates osteoclast differentiation, maturation, activation and survival, resulting in enhanced bone resorption and subsequent bone loss. OPG, acting as a decoy receptor, competes with RANK for RANKL binding and effectively controls RANKL/RANK signaling. OPG exerts biological effects that oppose those mediated by RANKL, including inhibition of late-stage osteoclast differentiation, activation of matrix osteoclast suppression and promotion of osteoclast apoptosis. The balance between RANKL and OPG, as reflected by their ratio, is critical for maintaining physiological bone formation and turnover, with a higher RANKL/OPG ratio favoring increased bone resorption [16]. Serum OPG levels 3 months (*p* < 0.001), 6 months (*p* < 0.001) and 12 months (*p* < 0.001) after LDLT were all lower than preoperative baseline (Figure 12). No apparent intra-group differences in RANKL (Figure 13) and OPG/RANKL ratio (Figure 14) were noted (*p* = 0.744 and *p* = 0.491, respectively).

**Figure 15:**
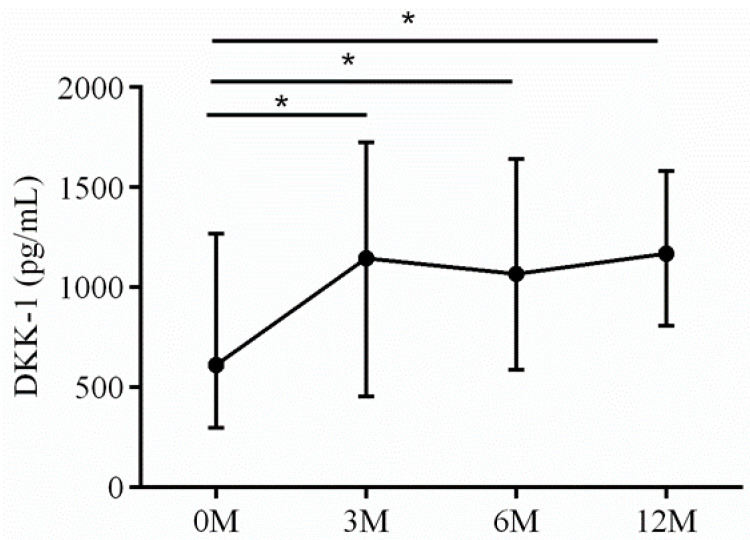
The changes in serum DKK-1 before and after LDLT. Friedman test *p* value: 0.006. Post hoc *p* values: 0M/3M 0.028, 0M/6M 0.010, 0M/12M 0.012. * *p* < 0.05.

Dickkopf-related protein 1 (DKK1) is a strong skeletal-deleterious factor [17]. DKK1 is a secreted protein that functions as an inhibitor of the canonical Wnt signaling pathway. It exerts its skeletal-deleterious effects by binding to the low-density lipoprotein receptor-related protein 5/6 (LRP5/6) on the surface of osteoblasts. The DKK1-LRP5/6 binding blocks the Wnt signaling pathway, resulting in the suppression of osteoblast proliferation and differentiation. Furthermore, DKK1 reduces OPG expression, thereby enhancing the biological effects of RANKL in inducing osteoclast differentiation [18]. The 3 month (*p* = 0.028), 6 month (*p* = 0.010) and 12 month (*p* = 0.012) serum DKK-1 levels were all higher than preoperative baseline (Figure 15).

Table 2 presents a comprehensive overview of the significant findings related to the alterations in lumbar spine and femoral neck T scores, as well as serum bone turnover markers, both before and after LDLT. All the ELISA findings mentioned above exhibited inter-assay percent coefficients of variability (% CVs) below 15 and intra-assay % CVs below 10. We also tried to determine the association between serum bone turnover markers and L-spine and femoral neck T score (Table 3). There was no apparent correlation between the T score of the L-spine and all the markers tested. However, the serum BAP (ρ = 0.221, *p* = 0.032) and CTX (ρ = 0.446, *p* < 0.001) had positive correlation with femoral neck T score, and serum 25-OH-D (ρ = −0.302, *p* = 0.003) and Ca (ρ = −0.330, *p* = 0.001) had negatived correlation with femoral neck T score.

Previous study has demonstrated that the interval between liver transplantation and hip fracture was 2.6  ±  2.7 years, so we followed our cohort for three years concerning the occurrences of hip or spine fractures [19]. During the three-year follow-up period, none of the participants necessitated liver biopsy, while six patients experienced the occurrence of new-onset bony fractures. We tried to compare lumbar spine and femoral neck T score and serum bone turnover markers between the LDLT patients with and without fracture. We showed that compared with the LDLT patients without fracture, the LDLT patients with fracture showed higher serum OPG (*p* = 0.046), lower serum RANKL (*p* = 0.001), a higher serum OPG/RANKL ratio (*p* < 0.001) and higher serum DKK-1 (*p* = 0.020) (Table 4).

We constructed receiver operating characteristic (ROC) curves to evaluate the discriminative potentials of preoperative OPG, RANKL, OPG/RANKL ratio and DKK-1 to differentiate the LDLT patients with and without hip or spine fracture 3 years after LDLT. For the differentiation between LDLT patients with and without fracture, the areas under the curve (AUC) for OPG, RANKL, OPG/RANKL ratio and DKK-1 were 0.78 ± 0.10, 0.96 ± 0.04, 1.00 ± 0.00 and 0.82 ± 0.08, respectively (Table 5).

Finally, we wanted to ascertain whether individuals who underwent LDLT and experienced fractures exhibited serum biomarker values at 3 months, 6 months and 12 months that could still be effectively differentiated using the baseline value-derived cut-off points (Table 6). Notably, patients who suffered from fractures consistently exhibited an OPG/RANKL ratio greater than 0.17 from the baseline assessment throughout the 12-month period following LDLT.

## 4. Discussion

Osteoporosis is a well-known risk following LDLT, which can result in hip or spine fractures, and the underlying causes are complex and not yet fully understood. Our study found a significant decrease in both lumbar spine and femoral neck T scores after LDLT, accompanied by a decrease in bone resorption markers TRAP and CTX, and an increase in liver regeneration markers IGF-1 and 25-OH-D. While serum calcium levels increased significantly, serum OPG decreased and DKK-1 increased. We also found that serum BAP and CTX showed a positive correlation, while serum 25-OH-D and Ca showed a negative correlation with femoral neck T score. Comparing patients who developed fractures within 3 years of LDLT to those who did not, we observed higher serum OPG, lower serum RANKL, a higher serum OPG/RANKL ratio and higher serum DKK-1 levels in the former. Furthermore, we found that OPG, RANKL, OPG/RANKL ratio and DKK-1 levels before LDLT had prognostic value for hip or spine fractures within 3 years after LDLT. Our study suggests that LDLT may contribute to decreased bone mass and bone resorption while promoting liver regeneration, and that serum OPG, RANKL, OPG/RANKL ratio and DKK-1 can serve as predictors of fractures after LDLT. These findings highlight the significance of serum bone turnover markers in predicting osteoporotic fractures after LDLT.

One Swedish group prospectively recruited 46 liver transplantation recipients [20]. The median bone loss over femoral neck 3 months post-transplant was 8.5%, and BMD declined slightly from 3 to 12 months following transplantation and rebound thereafter. The early bone loss was positively correlated with an increase in s-1-collagen-C-terminal telopeptide (CTX in our study) and urine N-terminal telopeptides. Bone formation markers increased slowly from 6 months post-transplant and onwards. Serum CTX demonstrated different association patterns in the Swedish study and ours. The reason for the discrepancy may be due to the substantially high HBV carrier rate in our cohort, as well as the notable difference in ethnicity between the two groups.

DKK-1 operates as an antagonist to the Wnt/β-catenin signaling pathway, and the use of antibodies targeting DKK-1 shows potential for the treatment of osteoporosis [18,21,22]. The role of the Wnt pathway in mediating bone diseases, including glucocorticoid use and estrogen deficiency-related bone loss, is well-demonstrated in previous studies [23]. Wnt/β-catenin signaling is also crucial for the development, regeneration and maintenance of normal physiologic functions as well as the pathologic processes of the liver [24]. Overexpression of DKK-1 has been shown to predict poor prognosis for patients with hepatocellular carcinoma after liver transplantation by promoting cancer metastasis and recurrence [25]. DKK-1 promotes angiogenesis and is a biomarker for hepatic stem cell-like hepatocellular carcinoma [26]. Our study revealed a significant increase in serum DKK-1 levels after LDLT, and we found that baseline levels could predict osteoporotic fractures within 3 years after the procedure. We hypothesize that elevated DKK-1 levels after LDLT may indicate improved allograft liver function, which could partially contribute to decreased BMD by inhibiting Wnt/β-catenin signaling.

The RANK/RANKL/OPG pathway is an important cytokine system in mediating osteoclastic differentiation [9,27]. RANK is the cell membrane receptor expressed by osteoclasts, and RANKL is the cell membrane ligand expressed by osteoblasts. The association of RANK with RANKL leads to osteoclastic differentiation and proliferation. OPG is the decoy receptor of RANK secreted by osteoblasts, and suppresses osteoclastic activities by blocking the RANK-RANKL association. Monegal et al. published the observation that cirrhotic patients with low bone mass had higher OPG serum levels than the control subjects [28]. Fabrega et al. reported that serum OPG and RANKL levels were both higher than the normal control at 1, 7 and 14 days after liver transplantation [29]. The authors attributed the elevation of RANKL to the activation of the immune system towards the allograft liver, with subsequent OPG elevation as an innate compensatory mechanism for rescuing the detrimental effects of RANKL over bone metabolism [29]. Our study showed that serum OPG levels at 3 months (*p* < 0.001), 6 months (*p* < 0.001) and 12 months (*p* < 0.001) after LDLT were significantly lower than the preoperative baseline. We also paradoxically found that OPG, RANKL and OPG/RANKL ratio had good discriminative power in differentiating LDLT patients with and without fractures, with an AUC of 0.78 ± 0.10, 0.96 ± 0.04 and 1.00 ± 0.00, respectively. Patients who developed osteoporotic fractures after LDLT had higher baseline OPG, lower baseline RANKL and a higher OPG/RANKL ratio. A recent meta-analysis suggested that bisphosphonate therapy could reduce fracture rates in liver transplant recipients, but the fracture incidence was still as high as 6.6% even in the bisphosphonate group [30]. Based on our findings and published studies, we hypothesize that a relatively low but not extremely low systemic osteoclast activity level may be optimal to prevent osteoporotic fractures in LDLT recipients.

In comparison to patients without fractures, those who experienced fractures after LDLT displayed higher levels of OPG, lower levels of RANKL, a higher OPG/RANKL ratio and elevated levels of DKK-1 before LDLT. The panoramic pattern did not exhibit significant alterations following LDLT. Based on these observations, we hypothesize that osteoclastic overactivity may not provide a plausible explanation for the occurrence of fractures. Instead, it appears that fine-tuning osteoclastic activities within an optimal range, rather than extremely high or low levels, is essential. Moreover, DKK-1, a factor detrimental to bone health, demonstrated higher levels in fracture patients, with a substantial increase observed after LDLT. These consistent findings highlight the potential of DKK-1 inhibition as a more straightforward approach for the treatment of bone disease following LDLT.

Contrary to the heightened vulnerability of elderly females to postmenopausal osteoporotic fractures, it is intriguing that the entire cohort consisted of only five females, none of whom encountered fractures. We postulate that the female LDLT patients in our cohort were relatively younger, spanning ages between 32 and 56 years, which only slightly overlapped with the age range associated with postmenopausal osteoporosis. The effects of allograft liver transplantation and immunosuppressive agents seemed to exert a more pronounced impact than the mechanisms underlying senile osteoporosis.

The primary limitation of our study lies in the small sample size. Nonetheless, the feasibility of enrolling additional participants who have undergone LDLT and voluntarily abstain from the usage of antiresorptive agents (such as bisphosphonate and denosumab) and/or bone formation agents (such as teriparatide) is highly challenging due to the widespread availability and accessibility of these therapeutic interventions. As a result, it is difficult to expand the sample size by including individuals who are willing to refrain from osteoporotic regimens both prior to and for a period of 3 years following LDLT. Secondly, it is unfeasible to ascertain the isolated impact of allograft liver on bone turnover, as all recipients were administered immunosuppressive agents, which have the potential to influence bone metabolism. All participants underwent DEXA assays and blood sampling exclusively at the baseline assessment, as well as at 3, 6 and 12 months following LDLT. Regrettably, the specific DEXA findings and blood samplings during the occurrence of fractures were not available for analysis.

## 5. Conclusions

Our study showed a significant decrease in lumbar spine and femoral neck T scores after LDLT. This decrease was accompanied by a decrease in bone resorption markers and an increase in liver regeneration markers. Serum calcium levels increased, while serum OPG decreased and DKK-1 increased. We found a positive correlation between serum BAP and CTX, while serum 25-OH-D and Ca showed a negative correlation with femoral neck T score. Patients who developed fractures within 3 years of LDLT had higher serum OPG, lower serum RANKL, a higher serum OPG/RANKL ratio and higher serum DKK-1 levels compared with those who did not. OPG, RANKL, OPG/RANKL ratio and DKK-1 levels before LDLT were predictive of hip or spine fractures within 3 years after LDLT. Based on our findings, we hypothesize that precise modulation of osteoclastic activities within an optimal range, as well as targeted inhibition of DKK-1 activity, holds promise as a therapeutic approach for managing bone disease after LDLT.

## Figures and Tables

**Table 1:** Baseline demographic data for the patients undergoing LDLT.

	Sex	Age	Etiology	Fracture after LDLT (within 3 Years)
1	F	32	PLD	
2	M	40	ALC	
3	M	43	HBV, HCC	T12/L1 VCF (12 months)
4	F	43	HBV	
5	M	44	HCV, HCC	
6	F	46	HBV	
7	M	46	HBV	
8	M	47	HBV, HCC	T12/L1 VCF (12 months)
9	M	48	HBV, HCC	
10	M	48	HCV, ALC	
11	M	50	HBV, HCV	L4 VCF (15 months)
12	M	51	ALC	L5 VCF (24 months)
13	F	51	HBV	
14	M	53	HBV, HCV, HCC	
15	M	54	HCV	
16	M	54	HCV	T12/L1 VCF (15 months)
17	M	56	HCC, ALC	
18	F	56	HBV, HCC	
19	M	57	HBV, HCC	
20	M	57	HBV	T12 VCF (24 months)
21	M	60	HCV, HCC	
22	M	58	HCV	
23	M	65	HBV	
24	M	67	HCV, HCC	
25	M	66	HBV, HCC	

PLD: polycystic liver disease; ALC: alcoholic liver cirrhosis; HBV: hepatitis B virus infection; HCV: hepatitis C virus infection; HCC: hepatocellular carcinoma; VCF: vertebral compression fracture.

**Table 2:** Alterations in lumbar spine and femoral neck T scores as well as serum bone turnover markers before and after LDLT.

	0M	3M	6M	12M	*p* Value
LS T score	−1.30 (−1.60, −0.80)	−1.20 (−2.00, −0.90)	−1.50 (−2.00, −1.10)	−1.40 (−2.00, −0.90)	0.024
FN T score	−1.30 (−1.80, −0.50)	−1.60 (−2.00, −0.90)	−1.60 (−1.90, −1.10)	−1.70 (−1.90, −1.00)	<0.001
BAP	24.86 (15.76, 27.83)	21.25 (18.39, 27.60)	21.64 (17.76, 28.48)	20.55 (12.38, 31.03)	0.577
OCN	5.83 (5.15, 7.65)	7.08 (6.06, 11.30)	7.88 (6.17, 13.32)	6.52 (6.06, 8.74)	0.082
TRAP	4.10 (3.31, 5.18)	2.38 (1.42, 3.57)	2.57 (1.63, 3.17)	2.98 (2.31, 4.11)	<0.001
CTX	0.76 (0.44, 1.09)	0.57 (0.34, 0.83)	0.39 (0.27, 0.59)	0.37 (0.23, 0.64)	0.001
IGF-1	84.76 (34.52, 107.29)	243.63 (157.45, 272.90)	190.07 (165.51, 248.58)	114.69 (889.30, 149.10)	<0.001
25-OH-D	16.00 (13.60, 20.90)	24.10 (21.40, 28.10)	23.80 (21.70, 29.70)	25.40 (18.80, 29.70)	0.001
iPTH	31.80 (14.80, 39.50)	34.80 (20.55, 44.65)	41.50 (30.95, 50.45)	36.90 (27.65, 46.20)	0.054
Ca	8.10 (7.60, 8.50)	8.90 (8.70, 9.30)	8.80 (8.65, 8.95)	8.80 (8.50, 9.00)	<0.001
P	3.20 (2.40, 3.90)	3.50 (3.20, 3.70)	3.20 (2.75, 3.55)	2.90 (2.60, 3.50)	0.045
OPG	8.47 (6.36, 10.74)	4.56 (2.87, 5.13)	4.41 (3.04, 5.61)	3.98 (2.77, 4.81)	0.002
RANKL	86.72 (53.54, 163.89)	61.53 (24.20, 162.53)	51.19 (28.36, 159.87)	55.91 (18.02, 206.27)	0.744
OPG/RANKL ratio	0.09 (0.05, 0.16)	0.05 (0.03, 0.20)	0.09 (0.03, 0.17)	0.04 (0.02, 0.29)	0.491
DKK-1	618.37 (300.64, 882.44)	1262.53 (650.08,1079.65)	1067.14 (599.35, 1713.41)	1096.26 (809.19, 1352.73)	0.006

**Table 3:** The Spearman’s correlation between serum bone turnover markers and L-spine and femoral neck T score.

	L-Spine		Femoral Neck	
	ρ	*p* Value	ρ	*p* Value
BAP	0.041	0.691	0.221	0.032
Osteocalcin	0.075	0.467	0.054	0.602
TRAP	0.102	0.325	0.133	0.196
CTX	0.164	0.110	0.446	<0.001
IGF-1	−0.020	0.848	−0.005	0.956
25-OH-D	−0.011	0.914	−0.302	0.003
iPTH	0.099	0.358	0.164	0.125
Ca	−0.033	0.751	−0.330	0.001
P	−0.165	0.283	0.122	0.242
OPG	0.050	0.655	−0.027	0.810
RANKL	−0.019	0.869	−0.099	0.378
OPG/RANKL ratio	0.014	0.898	0.066	0.562
DKK-1	0.057	0.589	−0.162	0.122

ρ: Spearman’s correlation coefficient.

**Table 4:** The comparison of lumbar spine and femoral neck T score and serum bone turnover markers between the LDLT patients with and without fracture.

	Fracture (+)	Fracture (−)	*p* Value
LS T score	−1.50 (−1.60, −1.10)	−1.30 (−1.55, −0.75)	0.424
FN T score	−1.75 (−2.10, −0.30)	−1.20 (−1.70, −0.55)	0.465
BAP	16.43 (14.44, 24.86)	25.01 (17.99, 31.98)	0.134
Osteocalcin	6.91 (5.38, 8.71)	5.72 (4.63, 7.11)	0.238
TRAP	3.73 (3.38, 4.08)	4.52 (3.22, 5.62)	0.194
CTX	0.55 (0.34, 0.63)	0.93 (0.52, 1.61)	0.080
IGF-1	80.79 (28.69, 88.42)	84.76 (35.99, 107.86)	0.631
25-OH-D	18.20 (14.30, 21.50)	16.00 (12.85, 19.85)	0.589
iPTH	38.50 (9.00, 39.90)	26.30 (14.80, 36.50)	0.298
Ca	8.45 (7.70, 8.80)	8.00 (7.55, 8.25)	0.171
P	2.85 (2.20, 3.90)	3.30 (2.50, 4.10)	0.749
OPG	10.88 (9.71, 11.45)	7.22 (6.31, 9.92)	0.046
RANKL	32.27 (29.93, 52.60)	104.75 (76.34, 189.34)	0.001
OPG/RANKL ratio	0.28 (0.24, 0.36)	0.08 (0.04, 0.11)	<0.001
DKK-1	1181.37 (930.30, 1705.89)	353.29 (260.95, 792.56)	0.020

Fracture (+): LDLT patients suffering from fracture within three-year follow-up; Fracture (−): LDLT patients without fracture within three-year follow-up.

**Table 5:** Area under the curve (AUC) of receiver operating characteristic (ROC) curve analyses for the items with significant differences between the LDLT patients with and without fracture in Table 3.

	Fracture (+) versus Fracture (−)		
	AUC	*p* Value	Cut-Off Value for Fracture
OPG	0.78 ± 0.10	0.042	>8.31
RANKL	0.96 ± 0.04	<0.001	<54.47
OPG/RANKL ratio	1.00 ± 0.00	<0.001	>0.17
DKK-1	0.82 ± 0.08	0.019	>661.35

Fracture (+): LDLT patients suffering from fracture within three-year follow-up; Fracture (−): LDLT patients without fracture within three-year follow-up.

**Table 6:** The number of individuals falling within the range defined by the cut-off value in the preoperative baseline and at 3, 6 and 12 months following transplantation.

	Cut-Off Value	0M		3M		6M		12M	
		(+)	(−)	(+)	(−)	(+)	(−)	(+)	(−)
OPG	>8.31	5/6	5/19	5/6	3/19	5/6	3/19	5/6	3/19
RANKL	<54.47	5/6	2/19	6/6	2/19	6/6	3/19	6/6	3/19
OPG/RANKL ratio	>0.17	6/6	0/19	6/6	0/19	6/6	0/19	6/6	1/19
DKK-1	>661.35	5/6	2/19	6/6	3/19	6/6	3/19	6/6	2/19

(+): LDLT patients suffering from fracture within three-year follow-up; (−): LDLT patients without fracture within three-year follow-up.

## Data Availability

The data presented in this study are available on request from the corresponding author.
